# Eco-Friendly Fiberboard Panels from Recycled Fibers Bonded with Calcium Lignosulfonate

**DOI:** 10.3390/polym13040639

**Published:** 2021-02-21

**Authors:** Petar Antov, L’uboš Krišt’ák, Roman Réh, Viktor Savov, Antonios N. Papadopoulos

**Affiliations:** 1 Faculty of Forest Industry, University of Forestry, 1797 Sofia, Bulgaria; victor_savov@ltu.bg; 2 Faculty of Wood Sciences and Technology, Technical University in Zvolen, 96001 Zvolen, Slovakia; kristak@tuzvo.sk (L.K.); reh@tuzvo.sk (R.R.); 3 Laboratory of Wood Chemistry and Technology, Department of Forestry and Natural Environment, International Hellenic University, GR-661 00 Drama, Greece

**Keywords:** wood-based panels, fiberboards, recycled fibers, bioadhesives, calcium lignosulfonate, zero-formaldehyde emission

## Abstract

The potential of using residual softwood fibers from the pulp and paper industry for producing eco-friendly, zero-formaldehyde fiberboard panels, bonded with calcium lignosulfonate (CLS) as a lignin-based, formaldehyde free adhesive, was investigated in this work. Fiberboard panels were manufactured in the laboratory by applying CLS addition content ranging from 8% to 14% (on the dry fibers). The physical and mechanical properties of the developed composites, i.e., water absorption (WA), thickness swelling (TS), modulus of elasticity (MOE), bending strength (MOR), as well as the free formaldehyde emission, were evaluated according to the European norms. In general, only the composites, developed with 14% CLS content, exhibited MOE and MOR values, comparable with the standard requirements for medium-density fiberboards (MDF) for use in dry conditions. All laboratory-produced composites demonstrated significantly deteriorated moisture-related properties, i.e., WA (24 h) and TS (24 h), which is a major drawback. Noticeably, the fiberboards produced had a close-to-zero formaldehyde content, reaching the super E0 class (≤1.5 mg/100 g), with values, ranging from 0.8 mg/100 g to 1.1 mg/100 g, i.e., equivalent to formaldehyde emission of natural wood. The amount of CLS adhesive had no significant effect on formaldehyde content.

## 1. Introduction

The resource efficiency optimization, and the transition to circular, low-carbon bioeconomy, have posed new requirements and actions towards a greater, more sustainable use of natural raw materials by sustainably increasing the primary production and conversion of waste into high-value added products. Cascading use of lignocellulosic resources, defined as “the efficient utilization of resources by using residues and recycled materials for material use to extend total biomass availability within a given system”, is one of the leading principles for achieving this goal.

The wood-based panel industry, with its wide variety of products for a number of end-uses, such as construction and furniture manufacturing, is one of the fastest growing industries worldwide, characterized by a clear upward trend for many years [1,2,3]. In 2019, the annual global production of wood-based composites was estimated to be 357 million m^3^, which represents an increase of 268% compared to 1980 [4]. The possibilities to reduce the increased consumption of wood raw materials include recycling of waste wood-based panels at the end of their life cycle [5,6,7,8,9,10], utilization of alternative raw materials [11,12,13,14,15], and use of waste lignocellulosic materials [16,17,18].

Global pulp, paper, and paperboard production is also increasing, and in 2019, it was estimated to approximately 202 million tons, and 404 million tons, respectively, with USA and China being the major producers [4]. Consequently, pulp and paper facilities generate substantial amounts of non-hazardous sludge and solid waste, requiring further waste management or utilization as by-products [19,20]. Currently, the main methods of disposal of the primary sludge are landfilling or burning for energy generation. However, due to the stricter environmental legislation and increased landfilling costs in the EU, the industry is searching for alternative waste management methods.

Most of the suspended solids are removed during primary mechanical wastewater treatment, and the resulting liquid sludge contains large quantities of residual wood fibers as the main organic components [19,21], thus representing a potential feedstock for manufacturing wood-based panels [22,23,24]. The residual fibers are significantly different in composition, even between factories using the same pulp and paper production technologies [25]. In addition, another distinct disadvantage for their wider utilization as a raw material is the significantly reduced lignin content [26].

Traditional synthetic adhesives, used in the production of wood-based panels, are usually made from petroleum-derived components, based on urea, formaldehyde, phenol, melamine, etc. [27,28,29,30]. Nowadays, approximately 95% of the total adhesives used for manufacturing wood composites, are formaldehyde-based resins [31], with urea-formaldehyde resins being the most predominant type, with an estimated global consumption of 11 million tons/year [32,33,34]. Despite their numerous advantages of conventional synthetic thermosetting adhesives, such as excellent adhesion properties and water resistance, ease of handling, low curing temperature, short press times, relative cost-effectiveness, etc. [33,35,36,37,38,39], they have a major drawback, connected to the hazardous emission of free formaldehyde and other volatile organic compounds (VOC) from the finished wood-based panels [40,41,42], associated with environmental problems and a number of serious human health hazards, such as such as eye, skin, and nervous system irritation, skin sensitization, nausea, and even cancer [43,44,45]. In 2004, formaldehyde was reclassified as “carcinogenic to humans” (Group 1) by the International Agency for Research on Cancer [46]. This has led to the adoption of stricter legislative regulations on formaldehyde emission values from wood-based panels, which have gradually been lowered over time, resulting in increased consumer environmental awareness [47] and greater industrial interest towards the development of less toxic, eco-friendly wood-based composites, where the traditional thermosetting resins have been partly or completely replaced by renewable, bio-based adhesives [48,49,50,51,52,53,54,55,56], or by adding different organic [57,58,59] and inorganic [40,44,48,60,61,62,63] compounds to adhesives systems as formaldehyde scavengers. Another possible solution to avoid the negative effect of formaldehyde release from wood-based panels is to use isocyanate adhesives, namely polymeric 4,4‘-diphenyl methane diisocyanate (pMDI), where no formaldehyde is added [33,64]. However, the relatively higher cost of pMDIs compared to the common formaldehyde-based adhesives and the need to adjust the glue lines [65] are the main limiting factors for their wider application as wood adhesives.

While the wood-based panel industry, mainly for reasons of supply, is still dominated by the traditional oil-derived adhesives, both in these fields as well as in the strongly upcoming field of bio-based adhesives, there has been almost incredible progress as well as developments dictated by the intellectual ferment induced by a number of outside constraints. These are the stricter government regulations to reduce and even eliminate formaldehyde and other materials that are to some extent toxic, consumer awareness and the consequent drive of industry to favor more environment-friendly materials and, finally, the drive of industry to decrease or even eliminate their dependence on petrochemicals, due to the real or imagined future decrease of oil reserves with its consequent increase in the price of raw materials for purely traditionally manufactured wood binders [66,67,68].

Recent developments in the field of sustainable, bio-based adhesives include the use of different renewable biomass feedstocks, such as proteins [69,70,71,72,73], starch [74,75], tannins [76,77,78], and lignin [17,79,80,81,82,83,84]. The development of alternative value-added wood composites that use waste materials or recycled materials is becoming beneficial due to over exploitation of natural resources. Nowadays, the products manufactured from recycled materials or by-products are especially paid attention in the view-point of environmental problems [85,86,87,88,89]. In this context, a good and representative example is the utilization of waste polystyrene as a binder in wood composites manufacture. This approach of retraining wood or wastes of wood and polystyrene obtained from packing remove from service permits to produce economic and environment respectful composites. Waste polystyrene poses serious environmental risks, especially in developing countries where disposal facilities are lacking and its management is a serious problem because it is easy to recycle. Its application as a binder in order to produce value-added wood composites avoids the environmental problems that formaldehyde adhesives cause. Masri et al. [90] successfully produced particleboards from date palm and expanded polystyrene (EPS) wastes and reported that the bending strength and stress reached acceptable values of 0.78 GPa and 2.84 MPa, coupled with good fiber-matrix interface adhesion. Akinyemi et al. [91] presented the results of the experimental study on the production of particle boards from wastes of wood and expanded polystyrene foam. This study demonstrated that wood and expanded polystyrene foam wastes are sustainable materials for producing composite wood-based panels that are still durable in a moist environment. Polystyrene has been also applied in the manufacture of lightweight gypsum-based composites [92].

Lignin is a highly-branched, polyaromatic macromolecule and the second most abundant organic material on earth after cellulose [93,94]. The structure of lignin is composed of different units and repeated structures, depending on the plant species, growing conditions, and duration, and the extraction method applied to separate lignin from hemicellulose and cellulose [95]. Currently, lignin is regarded as a waste or a by-product from the production of pulp, paper, and ethanol with an approximate annual production of 100 million tons worldwide [96], of which less than 2% is used for value-added applications, such as surfactants, adhesives, polymer reinforcement materials, dispersants, etc., while the rest is burnt for heat and energy [51,96,97]. Lignin contains different functional groups, i.e., hydroxyl, methoxyl, and carbonyl groups, which allow its chemical modification, applied to increase its reactivity. The polyphenolic structure of lignin is the main reason for its application in the composition of adhesives, mostly as a partial substitution of phenol (C_6_H_6_O) in phenol-formaldehyde resins [98].

The different technological processes, used in the pulp and paper industry to obtain lignin, include mechanical, chemical, and enzymatic methods, which subsequently produce different types of technical lignin, e.g., hydrolytic lignin obtained by enzymatic hydrolysis process [99], organosolv lignin by organosolv treatment [100], alkali lignin, derived by the Kraft process [101], and lignosulfonates, obtained by the sulfite pulping process [101]. Lignosulfonates (LS), produced by the reaction of sulfurous acid (H_2_SO_3_) and a sulfite (SO_3_^2−)^ or bisulfite salts (HSO_3_^2−^), comprising ammonium, sodium, calcium, or magnesium at varied pH levels [102], are available in large quantities, with an estimated global annual production of 1 million tons [103]. Due to the availability of sulfonate group, LS are water soluble. The relatively high molecular weight of LS, ranging from 1000–150,000 g·mol^−1^ [103], is another important factor affecting the performance of LS in adhesive applications. Therefore, additional chemical modification of LS, such as hydroxymethylation, can be applied to increase their reactivity [102,103]. The major drawbacks for the wider industrial application of LS in the composition of wood adhesives is the increased hydrophilicity of finished wood-based panels and longer press times [17,81,89], which can be resolved by using suitable cross-linkers [79,104] or by optimizing the production parameters [105].

The aim of this research work was to investigate the potential of producing eco-friendly fiberboard composites from residual wood fibers from the pulp and paper industry, bonded with formaldehyde-free adhesive, namely calcium lignosulfonate, complying with the European standards.

## 2. Materials and Methods

Residual fiber mass, a mixture of the softwood species Scots pine (*Pinus silvestris* L.) and Norway spruce (*Picea abies* Karst.), oven-dried to 11% moisture content, was used in this work. The industrial waste fibers were supplied by the Bulgarian pulp and paper factory Mondi Stambolyiski EAD. The fiber mass, comprised of untreated, broken wood fibers and fine cellulose fibrils, had a bulk density of 28.49 kg·m^−3^. The residual fibers had lengths from 520 to 1150 µm and a reduced lignin content of approx. 7% (factory data).

Calcium lignosulfonate (CLS) at 8%, 10%, 12%, and 14% (based on the dry weight of fibers) was used as a binder. The CLS (lignosulfonic acid calcium salt, CAS No. 8061-52-7) additive used was in the form of amorphous yellow-brownish water-soluble powder with the following characteristics: total solids content: 93%; calcium content: 6%; reduced sugars: 7%; ash content: 6%; acidic factor in 10% solution: pH = 4.3 ± 0.8; % volatiles by weight: 4–7 (water); bulk density: 0.55 g/mL. CLS was used as a water solution at 40% working concentration.

Commercially available urea-formaldehyde (UF) resin with a solids content of 64%, density of 1.29–1.31 g.cm^−3^ at 20 °C, pH value of 8.5, and a molar ratio (MR) of 1.16, was provided by the factory Kastamonu Bulgaria AD (Gorno Sahrane, Bulgaria).

**Figure 1:**
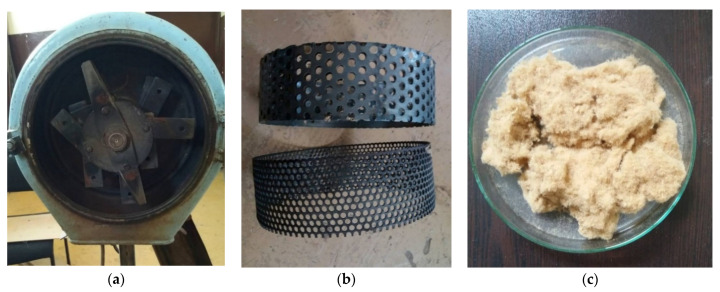
Prototype hammer mill used for preliminary treatment of residual wood fibers: (**a**) overview of prototype hammer mill; (**b**) hammer mill sieves, size 6 mm and 3 mm; (**c**) residual fibers after pretreatment.

Since the waste fibers were separated in a moist condition and contained a great number of cellulosic fibrils, they had to undergo a preliminary treatment before manufacturing the fiberboard composites. The pretreatment of waste fibers was performed by using a laboratory hammer mill (prototype, University of Forestry, Sofia, Bulgaria), shown in Figure 1.

After the initial treatment, the bulk density of residual fibers was decreased to 17.47 kg.m^−3^.

Fiberboard panels were manufactured with dimensions 400 mm × 400 mm, a thickness of 6 mm, and a target density of 750 kg.m^−3^. Four addition levels (8%, 10%, 12%, and 14%) of CLS as a binder, based on the dry fibers, were applied.

A control panel was produced with 10% UF resin content, based on the weight of dry fibers, and without CLS (panel REF10). This UF resin addition level (10%) is typical for the commercially produced fiberboard panels. The UF resin was used at 50% working concentration.

The manufacturing parameters of the laboratory-produced fiberboard panels are presented in Table 1.

Waste softwood fibers were mixed with the CLS additive in a high-speed glue blender with needle-shaped paddles (prototype, University of Forestry, BG) at 850 min^−1^. The CLS or UF resin was sprayed in the laboratory blender through a 1.5 mm nozzle, followed by injecting a paraffin emulsion. The hot pressing process was carried out using a single opening hydraulic press (PMC ST 100, Italy). The press temperature was 200 °C. The following four-stage pressing regime was applied: In the first stage, the pressure was increased to 4 MPa (15% of the press cycle); in the second stage, it was gradually decreased to 1.2 MPa (15% of the press time); in the third stage, the pressure was decreased to 0.8 MPa (60% of the press time). The fourth pressing period was performed at a pressure of 1.5 MPa (10% of the press time). The press factor applied was 60 s.mm^−1^. Following the hot pressing, the fiberboard panels were conditioned for 7 days at 20 ± 2 °C and 65% relative humidity.

**Figure 2:**
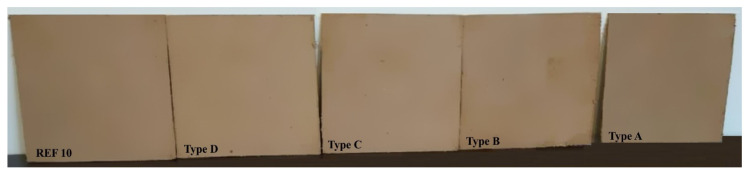
Fiberboard panels from residual softwood fibers from the pulp and paper industry, bonded with CLS; 750 kg·m^−3^ target density, 6 mm thickness, and four addition levels of ammonium lignosulfonate (8%, 10%, 12%, and 14%), and a control fiberboard panel (REF10), bonded with UF resin.

The physical and mechanical properties of the laboratory-produced panels (Figure 2) were determined in accordance with the European Standards EN 310, EN 317, EN 319, and EN 323 [106,107,108,109]. A precision laboratory balance Kern (Kern & Sohn GmbH, Balingen, Germany) with an accuracy of 0.01 g was used to determine the mass of the test samples. The dimensions of the test specimen were measured using digital calipers with an accuracy of 0.01 mm. The dimension stability (water absorption and thickness swelling) was measured after 24 h of immersion in water by the weight method [107]. The test samples were sanded before carrying out the tests. The mechanical properties (bending strength and modulus of elasticity) of the fiberboard panels were measured using a universal testing machine Zwick/Roell Z010 (Zwick/Roell GmbH, Ulm, Germany). For each property, eight test samples were used for testing.

The formaldehyde content of the laboratory-produced panels was measured in the factory laboratory of Kronospan Bulgaria EOOD (Veliko Tarnovo, Bulgaria) on four test specimens in accordance with the standard perforator method EN 124,650 [110].

Variational and statistical analysis of the results was performed by using the specialized software QstatLab 6.0. One-way analysis of variance (ANOVA) was carried out to discern significant difference at 95% level of confidence, using SAS software program (version 9.2, 2010) (SAS, Cary, NC, USA). Grouping was then made between treatments using Duncan’s multiple range test.

## 3. Results and Discussion

### 3.1. Physical and Mechanical Properties

The density of the laboratory-produced panels varied from 739 to 767 kg·m^−3^, rather close to the targeted value. The difference in this main characteristic of the fiberboards was significantly below 5%; thus, it did not have a significant effect on the other physical and mechanical properties of the panels.

**Figure 3:**
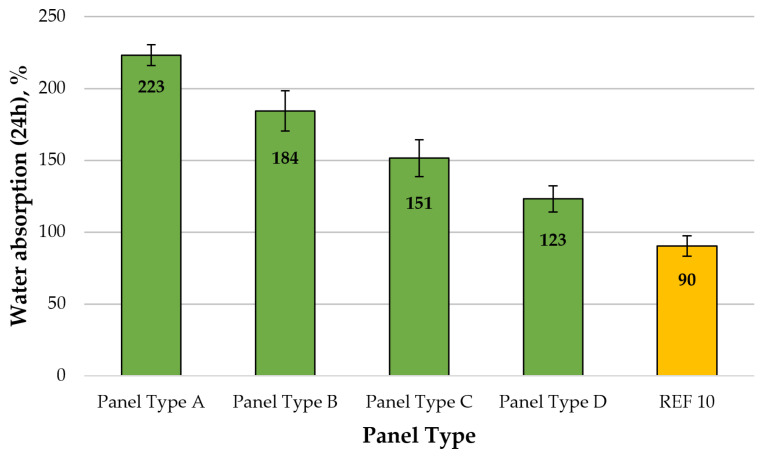
Water absorption (24 h) of fiberboard panels produced: type A—8% CLS; type B—10% CLS; type C—12% CLS; type D—14% CLS, and REF10—10% UF resin. (Error bar represents the standard deviation).

Water absorption (WA) and thickness swelling (TS) are important physical properties, strongly associated with the dimensional stability of wood-based panels [27,28,111]. Both properties were determined after 24 h immersion in water. A graphical representation of the WA (24 h) of the laboratory-made fiberboard panels is presented in Figure 3. WA is not a standardized physical property; nevertheless, the WA after 24 h of fiberboard panels, varied from 223% to 123%, i.e., a significant increase from approximately 148% in panel Type A (8% CLS) to 37% in panel Type D (14% CLS), compared with the WA of the control panel (REF10), was found. Therefore, CLS, as a lignin-based, formaldehyde-free adhesive, will require a further chemical modification to increase its adhesion efficiency [17,79,112]. Increasing the CLS content from 8% to 14% resulted in a gradual decrease of WA of the samples of approximately 49%.

Comparable WA values of approximately 170% were achieved by [17,81] for eco-friendly composites, comprised of recycled industrial wood fibers bonded with 15% content of magnesium lignosulfonate (MLS) as a binder, and veneered with beech veneers, also glued with MLS.

**Figure 4:**
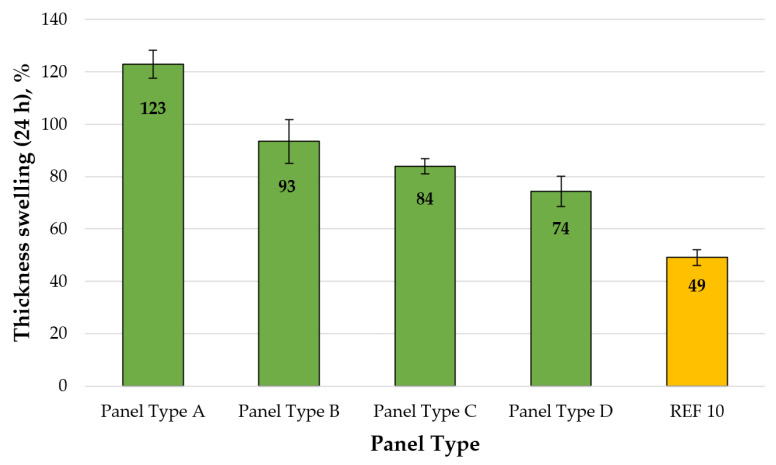
Thickness swelling (24 h) of fiberboard panels produced: type A—8% CLS; type B—10% CLS; type C—12% CLS; type D—14% CLS, and REF10—10% UF resin. (Error bar represents the standard deviation).

A graphical representation of the TS values (24 h) of the laboratory-produced fiberboard panels is shown in Figure 4.

As seen in Figure 4, the laboratory-produced fiberboard panels, composed of industrial waste fibers bonded with CLS adhesive, exhibited very high TS values, ranging from 123% to 74%, i.e., the values obtained were from 4.1 to 2.5 higher than the minimum limit to MDF panels for use in dry conditions (30%), required by the standard EN 622-5 [113]. Even the control panel (REF10), manufactured from residual wood fibers bonded with 10% UF resin, had TS values, 1.6 times higher than the standard requirements. These very high TS values may be attributed to the significantly reduced lignin content of residual fibers [17,81] and the characteristics of the CLS used as a bio-based adhesive [97,98]. The most significant improvement of TS values was determined when the CLS content was increased from 8% to 10%.

In terms of mechanical properties, the modulus of elasticity (MOE) and bending strength (MOR) were tested.

**Figure 5:**
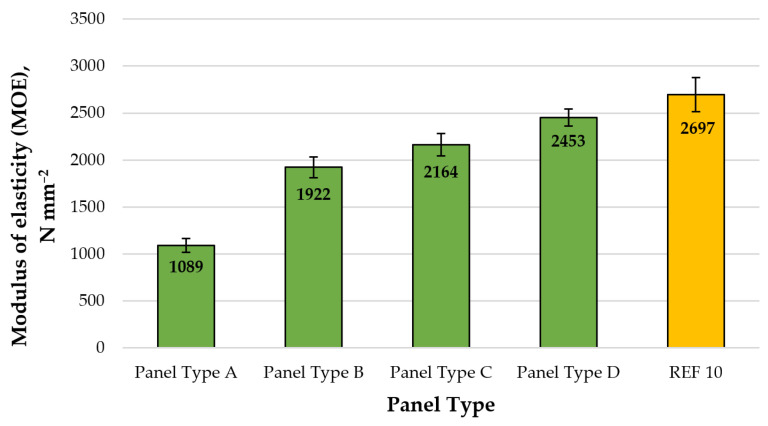
Modulus of elasticity (MOE) of fiberboard panels produced: type A—8% CLS; type B—10% CLS; type C—12% CLS; type D—14% CLS, and REF10—10% UF resin. (Error bar represents the standard deviation).

A graphical representation of the mean MOE values of the laboratory-fabricated fiberboard panels is presented in Figure 5.

The fiberboard panels, bonded with CLS as an eco-friendly adhesive, exhibited MOE values, ranging from 1089 to 2453 N.mm^−2^. The increased CLS addition from 8% to 14% resulted in improved MOE values by 125%. Similar results were reported by [17] in their research on the development of eco-friendly composites produced from recycled wood fibers bonded with 15% MLS as a binder. None of the experimental fiberboard panels met the European standard requirements EN 622-5 [113] for MDF panels used in dry conditions (≥2700 N.mm^−2^). The control fiberboard panel (REF10), fabricated with recycled fibers bonded with 10% UF resin, had 1.1 times higher MOE values than the panel, produced with 14% CLS as a binder (panel type D).

**Figure 6:**
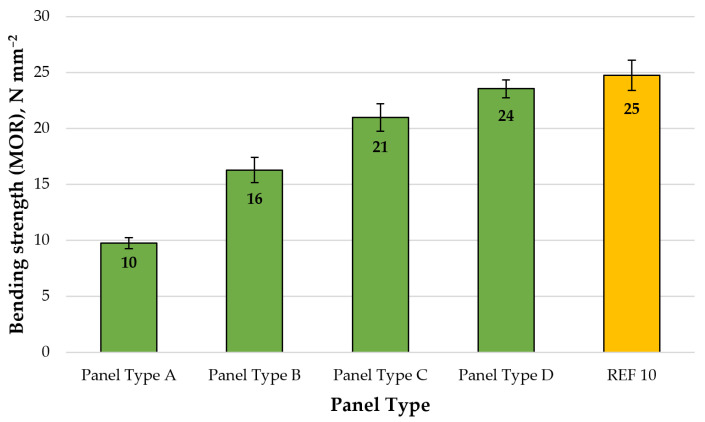
Bending strength (MOR) of fiberboard panels produced: type A—8% CLS; type B—10% CLS; type C—12% CLS; type D—14% CLS, and REF10—10% UF resin. (Error bar represents the standard deviation).

Finally, a graphical representation of the average MOR values of the laboratory-fabricated fiberboard panels is shown in Figure 6.

The fiberboard panels exhibited MOR values, ranging from 10 to 24 N.mm^−2^. The increased CLS content from 8% to 14% resulted in improved MOR values by 2.4 times. The most significant improvement by 60% was determined when the CLS content was increased from 8% to 10%. The panels bonded with 14% CLS (panel type D), met the standard requirement for MDF panels used in dry conditions (23 N.mm^−2^) [113]. The maximum MOR value obtained in this work (24.8 N.mm^−2^), was determined for the control panel (REF10), bonded with 10% UF resin. The fiberboard panel, bonded with the highest addition level of CLS (14%), reached MOR values, relatively close to the control panel with a difference of 4% only. Antov et al. [98] concluded that MDF panels, complying with the European standard requirements, can be successfully produced with a very low phenol-formaldehyde (PF) gluing factor of 3.5% (based on the dry fibers) and CLS addition levels, varied from 5% to 15%. In the same research, the maximum MOR values (35.2 N.mm^−2^) were determined in MDF panels, fabricated with 5% PF resin and 5% CLS additive. This can be explained by the presence of phenolic and aliphatic hydroxyl groups in CLS, which increase the reactivity of lignin towards synthetic thermosetting resins [114].

Higher MOR and MOE values of the developed fiberboard panels could be achieved by covering their surfaces with veneers, melamine, high-pressure laminate, etc.

### 3.2. Formaldehyde Content

The results for the formaldehyde content of the laboratory-produced fiberboard panels, measured according the standard EN ISO 12460-5 (known as the perforator method) [110], are presented in Table 2.

It is clearly revealed from the data depicted in Table 2 that all types of panels bonded with CLS adhesive, namely type A to D, has low formaldehyde content and can be considered as zero formaldehyde content [30,104]. It is further reveled, that these values are significantly different with the value of the reference panel (REF10). Closer inspection of the data presented in Table 2, shows that the amount of CLS adhesive has no significant effect on formaldehyde content.

All laboratory-made fiberboard panels exhibited formaldehyde content values, fulfilling the requirements of the super E0 emission category (≤1.5 mg/100 g). The lowest free formaldehyde content of 0.8 ± 0.1 mg/100 g was determined for panel Type D, bonded with 14% CLS. The reference panel (REF10), manufactured from waste fibers bonded with 10% UF resin only, can be classified under the E1 emission grade (≤8 mg/100 g). The results achieved are in accordance with our previous studies, where using different types of lignosulfonates in adhesive formulations for wood-based panels resulted in remarkably low formaldehyde content of the finished composites [17,81,82,98]. This might be attributed to the high amount of reactive phenolic and hydroxyl groups in CLS, which increase its reactivity towards formaldehyde [97,98,114]. Taking into consideration that natural wood releases low, but still detectable amount of formaldehyde, caused by its main polymeric constituents (cellulose, hemicellulose, and lignin) and extractives of approximately 0.5 to 2 mg/100 g [115,116,117], allows for defining the produced fiberboard panels, bonded with CLS as markedly low-emission wood-based panels.

## 4. Conclusions

Eco-friendly fiberboard panels with acceptable physical and mechanical properties in accordance with the European standards, with the exception of TS, and extremely low formaldehyde content, may be manufactured from residual wood fibers from the pulp and paper industry, bonded with CLS as a formaldehyde-free, lignin-based adhesive, applied at the content of 8% to 14%, based on the dry fibers. The laboratory-fabricated panels, bonded with 14% CLS, exhibited MOR and MOE values, comparable with the minimum standard requirements for MDF panels used in dry conditions [113]. The formaldehyde content of the fiberboard panels bonded with various amount of CLS adhesive, tested in accordance with the perforator method [110], was remarkably low and significantly different with the value of the reference panel (REF10), ranging from 0.8 mg/100 g to 1.1 mg/100 g, i.e., equal to formaldehyde release of natural wood, which allowed for their classification as eco-friendly wood-based panels. It is also found that the amount of CLS adhesive has no significant effect on formaldehyde content. According to their mechanical properties, the developed eco-friendly composites may be used in dry conditions for non-load bearing applications or as decorative panels.

Nevertheless, the significantly deteriorated dimensional stability, i.e., WA and TS, evaluated after 24 h immersion in water, represents the main drawback of the laboratory-produced fiberboard panels. Future studies on using CLS in wood adhesive applications should be aimed at optimizing the production parameters, improving the CLS composition by adding appropriate cross-linkers, and extensively investigating the interaction processes between lignocellulosic fibers and lignosulfonate additives.

## Figures and Tables

**Table 1:** Manufacturing parameters of fiberboard panels, produced from residual softwood fibers, bonded with CLS.

Panel Type	Adhesive Type	Density (kg·m^−3^)	UF Resin Content (%)	Calcium Lignosulfonate Content (%)
Type A	CLS	750	0	8
Type B	CLS	750	0	10
Type C	CLS	750	0	12
Type D	CLS	750	0	14
REF10	UF	750	10	0

**Table 2:** Formaldehyde content of fiberboard panels, produced from residual softwood fibers bonded with CLS, according EN ISO 12460-5.

HDF Type	Adhesive	UF Resin Content (%)	Calcium Lignosulfonate Content (%)	Formaldehyde Content (mg/100 g)
Type A	CLS	0	8	1.1 (± 0.1) ^1^ A ^2^
Type B	CLS	0	10	1.0 (± 0.1) A ^2^
Type C	CLS	0	12	0.9 (± 0.1) A ^2^
Type D	CLS	0	14	0.8 (± 0.1) A ^2^
REF10	UF	10	0	6.8 (± 0.1) B ^2^

^1^ Standard deviation. ^2^ Groupings based on Duncan’s multiple range test, at 95% level of confidence.

## Data Availability

The data presented in this study are available on request from the corresponding author.
